# Evolving trends in dupilumab use for chronic rhinosinusitis with nasal polyps (CRSwNP): a 4-year cohort analysis

**DOI:** 10.1038/s41598-025-17787-4

**Published:** 2025-12-18

**Authors:** Patrick Huber, Moritz Gröger, Clemens Stihl, Hanna Frankenberger, Donata Gellrich

**Affiliations:** Department of Otorhinolaryngology, Head and Neck Surgery, LMU Klinikum, Ludwig Maximilian University of Munich, Marchioninistraße 15, 81377 Munich, Germany

**Keywords:** CRSwNP, Dupilumab, Chronic rhinosinusitis, Biologics, Biological therapy, Quality of life, Inflammatory diseases

## Abstract

Chronic rhinosinusitis with nasal polyps (CRSwNP) affects up to 4.5% of the population and is frequently driven by type 2 inflammation. Dupilumab, targeting IL‑4 and IL‑13 pathways, was approved in 2019 as the first biologic for CRSwNP in Germany. This single-center, retrospective study analyzed 174 patients initiating dupilumab between 2019 and 2023. Patients were grouped by year and assessed for trends in demographics, surgical history, and treatment response. Only adherent patients with ≥ 6 months of follow-up were included. Age and baseline disease severity were stable across cohorts. However, the proportion of patients with > 3 prior sinus surgeries declined from 28.3% in 2019/20 to 13.0% in 2023, suggesting earlier biologic initiation over time. All patients met at least three EPOS 2020 criteria. Significant and clinically meaningful improvements were observed in all cohorts for Nasal Polyp Score (NPS), SNOT‑22, VAS, and olfactory testing, with changes exceeding established minimal clinically important differences (MCIDs). No cohort showed superior outcomes. Tese findings highlight consistent real-world efficacy of dupilumab and evolving prescribing patterns that favor earlier intervention, while reflecting continued cautious patient selection in the context of high treatment costs.

## Introduction

Chronic rhinosinusitis with nasal polyps (CRSwNP) is a persistent inflammatory condition of the nose and paranasal sinuses, defined according to EPOS 2020 by the presence for ≥ 12 weeks of two or more symptoms, including nasal blockage/congestion/obstruction or nasal discharge, with or without facial pain/pressure or reductio/loss of smell, and bilateral polyps confirmed on endoscopy^1–3^. Affecting 0.5–4.5% of the general population^4,5^, CRSwNP presents a significant socio-economic burden, with an average age of diagnosis between 40 and 60 years^6^.

Advances in understanding chronic rhinosinusitis (CRS) pathomechanisms have led to an endotype-based classification system, as described in the European Position Paper on Rhinosinusitis and Nasal Polyps 2020 (EPOS 2020)^3^. Approximately 85% of CRSwNP cases in the western hemisphere show a predominant eosinophilic type 2 endotype^7^, which is often associated with other type 2 inflammatory diseases, such as asthma. This endotype is also linked to a higher likelihood of disease recurrence despite standard treatments, including topical and systemic corticosteroids and endoscopic sinus surgery (ESS), presenting ongoing challenges for both patients and clinicians^6,8^.

Type 2 inflammation is characterized by elevated cytokines, including IL-4, IL-5, IL-13, and IgE, along with increased levels of innate lymphoid cells (ILC2), macrophages, and mast cells^1^. Eosinophils are key players, contributing to tissue damage, allergic responses, and chronic inflammation by releasing pro-inflammatory mediators, interacting with immune cells, and promoting tissue remodeling^9^.

These insights have led to the development of biologic agents targeting specific cytokines and mediators of type 2 inflammation. Presently, three biologics have been approved in Germany as adjunctive therapies for uncontrolled CRSwNP. The inaugural and in our experience most commonly prescribed biologic approved for CRSwNP is dupilumab, authorized in late 2019^10^ later followed by omalizumab and mepolizumab^11^. Dupilumab selectively binds to the alpha subunit of the IL-4 receptor, an integral component of both the heterodimeric IL-4 receptor and the IL-13 receptor. Consequently, dupilumab impedes signaling pathways for both IL-4 and IL-13, effectively mitigating type 2 inflammation^12^.

Our group, along with others, has previously provided compelling evidence of dupilumab’s effectiveness in reducing CRSwNP burden and enhancing patient-reported outcomes in real-world settings^13–16^.

However, despite recent European cost studies on CRSwNP^17,18^, little is known about how patient selection and treatment strategies have evolved since the introduction of biologics. The objective of this study was not to reassess short-term treatment efficacy, but rather to investigate whether patient selection patterns, demographic profiles, and baseline clinical characteristics have changed since the clinical implementation of dupilumab.“Using consecutive yearly initiation cohorts from 2019 to 2023 at a tertiary referral center in Germany, we analyzed trends in surgical history, comorbidities, and disease severity. This design was based on the hypothesis that, over time, patients starting dupilumab would present earlier in their disease course—being younger, less surgically burdened, and potentially with fewer comorbidities—reflecting evolving prescription practices and adaptation within the healthcare system.

## Materials and methods

### Study design and patient population

This study was a single-center, retrospective analysis of real-world data from a tertiary referral center in Germany. It included adult patients (≥ 18 years) with CRSwNP, as defined by the EPOS2020 guidelines, who began biologic treatment with dupilumab alongside ongoing intranasal corticosteroid therapy. Patients were grouped by the year of treatment initiation: 2019/20, 2021, 2022, and 2023. The years 2019 and 2020 were combined, as dupilumab was only approved for use in Germany starting in October 2019. The study compared demographic characteristics, medical histories, and immunological profiles across these cohorts. Only patients with a follow-up period of at least six months post-treatment initiation as of July 2024 were included in the study. Also, only patients who demonstrated adherence to the prescribed dupilumab regimen throughout the observation period were included in the analysis.

The study conformed to the 1976 Declaration of Helsinki and was approved by the local ethics committee and the local data protection commissioner under the project number 22–0802. All patients gave formal consent for data collection and study participation.

### Treatment

Dupilumab (Dupixent^®^, Sanofi S.A.) administered as a 300 mg injection was self-administered subcutaneously following the guidelines provided by the manufacturer once every two weeks as an add-on treatment to continued use of intranasal corticosteroids.

### Data collection

Prior to initiation of dupilumab treatment extended medical history was collected including history of sinus surgeries, intake of oral corticosteroids (OCS), evidence of blood eosinophilia and existence of comorbidities within the spectrum of type 2 inflammatory diseases such as self-reported asthma, allergic rhinitis (defined as sensitization to ≥ 1 aeroallergen by serum specific IgE or skin prick test, with matching nasal/ocular symptoms), urticaria and atopic dermatitis.

At initiation of dupilumab treatment and at follow-up timepoints subjective and objective assessments, as well as total IgE levels and eosinophil counts as biomarkers related to the respiratory condition, were gathered. These included visual analogue scale (VAS) for overall disease impairment (recorded from 0–10 as indicated by the patient on a 10 cm line)^19^, Sinonasal Outcome Test − 22 questionnaire (SNOT-22 from 0-110), total endoscopic Nasal Polyp Score (NPS, as proposed by Gevaert et al.^20^ and van Zele et al.^21^: 0–4 on every side, total score 0–8), psychophysical olfactory testing, either by Sniffin’ Sticks 12-item identification test (SSIT-12) or University of Pennsylvania Smell Identification Test (UPSIT). Olfactory function was classified into anosmia (0–6), hyposmia (7–10) and normosmia (11–12) as described be Lawton et al.^22^.

Therapeutic response was classified according to the EPOS/EUFOREA update 2023 into good-excellent response, poor-moderate response and no response^23^.

### Statistical analysis and visualization

Shapiro-Wilk test was used to test the data for normality. To compare between baseline and individual post-intervention time points paired t-tests were used for normally distributed data and Mann-Whitney test was performed for not normally distributed data. To examine overall differences across multiple time points, the Kruskal-Wallis test was applied. All statistical analyses and data visualization were carried out using SigmaPlotTM 14.5 software tools (Systat Software, San Jose, USA). Unless otherwise indicated results are presented as mean ± standard deviation. A p-value of < 0.05 was considered to indicate statistical significance.

## Results

The study analyzes demographic and baseline characteristics of four patient cohorts with chronic rhinosinusitis with nasal polyps (CRSwNP) who started dupilumab treatment in the years 2019/20, 2021, 2022, and 2023. A total of 174 patients were included, distributed across these cohorts as follows: 60 patients in the 2019/20 cohort, 58 in 2021, 33 in 2022, and 23 in 2023.

Overall, the cohorts demonstrated substantial similarities in demographic and clinical profiles, including age, prevalence of comorbidities and baseline disease severity as shown in Table 1. Nevertheless, some distinct differences were observed, particularly in gender distribution, history of endoscopic sinus surgeries (ESS), and certain EPOS 2020 criteria.

**Table 1:** Demographics and baseline characteristics of the study population.

	2019/20		2021		2022		2023	
	*N* = 60		*N* = 58		*N* = 33		*N* = 23	
Age at start of therapy	49.3	(21–75)	47.6	(23–70)	49.4	(24–79)	49.7	(18–78)
**Sex**								
Male	36	(60.0%)	37	(63.8%)	20	(60.6%)	10	(43.5%)
Female	24	(40.0%)	21	(36.2%)	13	(39.4%)	13	(56.5%)
**Previous ESS**								
Min 1 ESS	60	(100.0%)	57	(98.3%)	33	(100%)	22	(95.6%)
> 3 ESS	17	(28.3%)	11	(18.9%)	5	(15.2%)	3	(13.0%)
> 5 ESS	6	(10.0%)	6	(10.3%	1	(3.1%)	1	(4.4%)
Time since last ESS (years)	4.4	(0–20)	6.4	(0–30)	4.44	(0–15)	6.5	(0–25)
Mean No. of surgeries	3.0	(1–8)	2.9	(0–15)	2.7	(0–7)	2.3	(0–6)
**Evidence of type 2 comorbidities**								
Asthma bronchiale	56	(93.3%)	43	(74.1%)	23	(71.9%)	18	(94.7%)
Allergic Rhinitis	32	(53.3%)	29	(50.0%)	12	(37.5%)	7	(36.8%)
Urticaria	2	(3.3%)	0	(0.0%)	0	(0.0%)	0	(0.0%)
Atopic dermatitis	1	(1.7%)	3	(5.2%)	0	(0.0%)	1	(5.3%)
**EPOS 2020 criteria**								
Evidence of type 2 inflammation	58	(100.0%)	56	(96.6%)	30	(90.1%)	22	(95.7%)
Need for OCS or contraindication	52	(89.7%)	50	(86.2%)	26	(78.8%)	22	(95.7%)
Significantly impaired HRQoL	58	(100.0%)	45	(77.6%)	31	(93.9%)	23	(100.0%)
Significant loss of smell	44	(75.9%)	45	(77.6%)	25	(75.8%)	19	(82.6%)
Diagnosis of comorbid asthma	56	(93.3%)	43	(74.1%)	23	(69.7%)	18	(78.3%)
**Number of EPOS2020 criteria met**								
1	0	(0.0%)	1	(1.7%)	0	(0.0%)	0	(0.0%)
2	0	(0.0%)	1	(1.7%)	1	(3.0%)	0	(0.0%)
3	8	(13.3%)	14	(24.4%)	7	(21.2%)	2	(8.7%)
4	21	(35.0%)	17	(29.3%)	12	(36.4%)	10	(43.5%)
5	31	(51.7%)	25	(43.1%)	13	(39.4%)	11	(47.8%)
≥3	60	(100.0%)	56	(96.6%)	32	(97.0%)	23	(100.0%)

Values are presented as the mean (range), unless stated otherwise. Percentages provided are computed based on the proportion of patients with available data. Cut-offs for EPOS 2020 biologic indication criteria: type 2 inflammation (tissue eosinophils ≥ 10 cells/HPF, blood eosinophils ≥ 250 cells/µL, or total IgE ≥ 100 IU/mL), need for systemic corticosteroids (≥ 2 courses/year, long-term low-dose use, or contraindication), significantly impaired HRQoL (SNOT-22 ≥ 40), significant loss of smell (anosmia on smell testing), and comorbid asthma (physician-diagnosed).

ESS, endoscopic sinus surgery; No, Number; EPOS 2020, European Position Paper on Rhinosinusitis and Nasal Polyps 2020; OCS, oral corticosteroids; HRQoL, health-related quality of life.

### Age and gender

Patients’ ages at the start of therapy demonstrated a stable age profile across the cohorts, with mean ages of 49.3 years (2019/20), 47.6 years (2021), 49.4 years (2022), and 49.7 years (2023). Gender distribution was predominantly male across most cohorts, with male patients constituting 60.0% (2019/20), 63.8% (2021), and 60.6% (2022). However, a notable shift occurred in 2023, where females represented 56.5% of the cohort.

### Medical history and surgical interventions

**Fig. 1:**
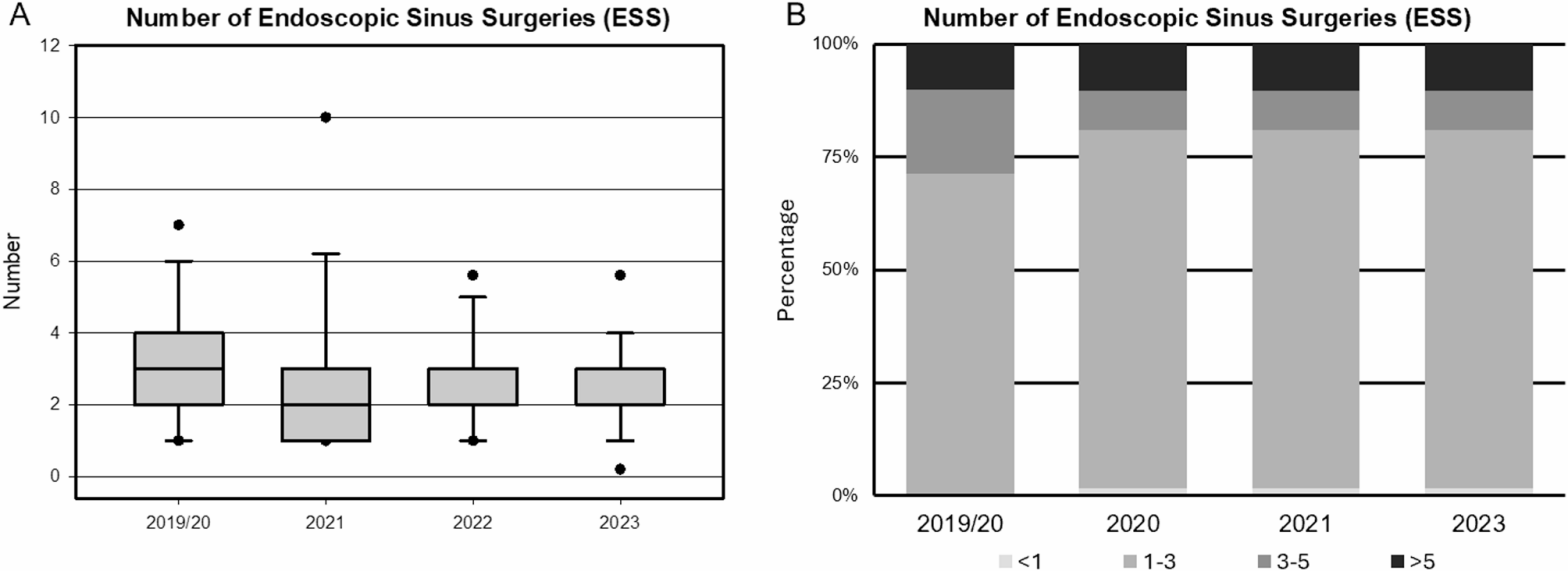
Boxplots (**A**) and Barplots (**B**) displaying the number of Endoscopic Sinus Surgeries (ESS) prior to biologic treatment initiation in different cohorts. Dots identifying 5–95% confidence interval.

All cohorts had a substantial history of ESS, reflecting consistent surgical backgrounds among patients with CRSwNP. In each cohort, nearly all patients had undergone at least one ESS procedure (100% in 2019/20, 98.3% in 2021, 100% in 2022, and 95.6% in 2023). Differences emerged in the extent of surgical history as depicted in Fig. 1B: patients with more than three prior ESS procedures decreased progressively from 28.3% in 2019/20 to 13.0% in 2023, indicating a reduction in high-frequency surgery patients in more recent cohorts. Similarly, the percentage of patients with more than five ESS procedures decreased, from 10.0% in 2019/20 and 2021 to 4.4% in 2023. The mean number of surgeries also exhibited a decreasing trend as shown in Fig. 1A, although not significant from 3.0 ± 3.9 in 2019/20 to 2.3 ± 1,25 in 2023, alongside variability in the average years since the last ESS, which ranged from 4.4 ± 4.8 years in 2019/20 to 6.5 ± 7.2 years in 2023.

### Type 2 inflammatory comorbidities

Type 2 inflammatory comorbidities were highly prevalent across all cohorts, particularly asthma, which was self-reported in 93.3% of patients in 2019/20, decreasing slightly to 74.1% in 2021 and 71.9% in 2022, before returning to 94.7% in 2023. Allergic rhinitis prevalence, while consistently observed across all years, exhibited a gradual reduction from 53.3% in 2019/20 to 36.8% in 2023. Other type 2 comorbidities, such as urticaria and atopic dermatitis, remained rare across the cohorts, with urticaria only appearing in 2019/20 (3.3%), and atopic dermatitis present only sporadically, peaking slightly in 2021 (5.2%) and 2023 (5.3%).

### EPOS 2020 criteria fulfillment

The EPOS 2020 criteria for initiating biologic treatment in patients with CRSwNP—including type 2 inflammation (tissue eosinophils ≥ 10 cells/HPF, blood eosinophils ≥ 250 cells/µL, or total IgE ≥ 100 IU/mL), corticosteroid requirements (≥ 2 courses/year or long-term low-dose use) or contraindication to them, impaired health-related quality of life (HRQoL) (SNOT-22 ≥ 40), and olfactory dysfunction (anosmia on smell testing) and and diagnosis of comorbid asthma—were consistently met across all cohorts, highlighting a comparable baseline clinical severity.

**Fig. 2:**
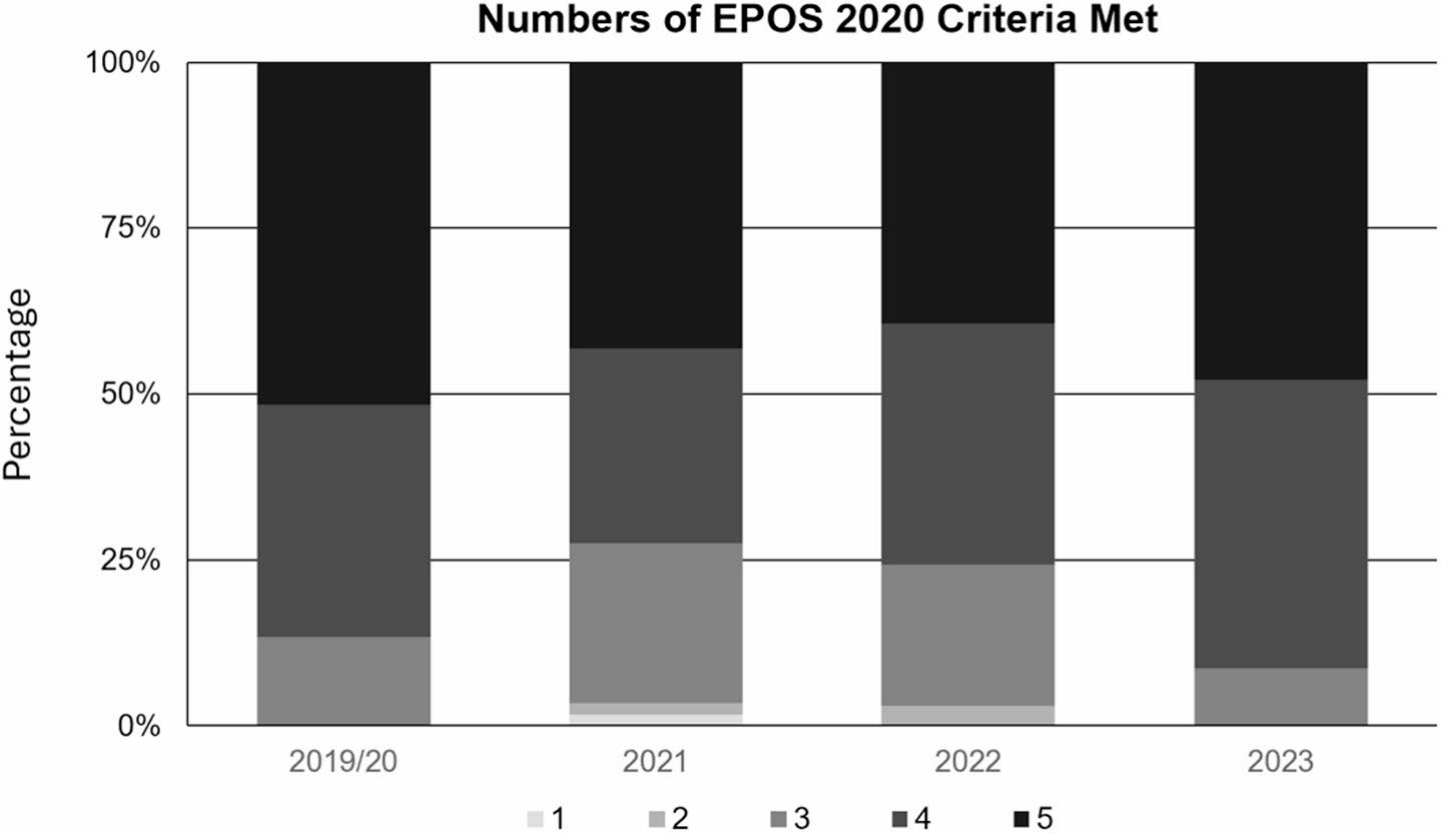
Barplots displaying the differences in numbers of EPOS 2020 criteria met in different cohorts. EPOS 2020, European Position Paper on Rhinosinusitis and Nasal Polyps 2020.

As illustrated in Fig. 2, a substantial proportion of patients in each year fulfilled multiple criteria, with consistent adherence to at least three criteria across all cohorts. In 2019/20, 100% of patients met at least three criteria; 35.0% fulfilled four, and 51.7% fulfilled all five. Similarly, in 2021, 96.6% met at least three criteria; 29.3% met four, and 43.1% met all five. In 2022, 97.0% fulfilled at least three criteria; 36.4% fulfilled four, and 39.4% fulfilled all five. In 2023, all patients met at least three criteria; 43.5% fulfilled four, and 47.8% fulfilled all five.

### Clinical parameters

**Fig. 3:**
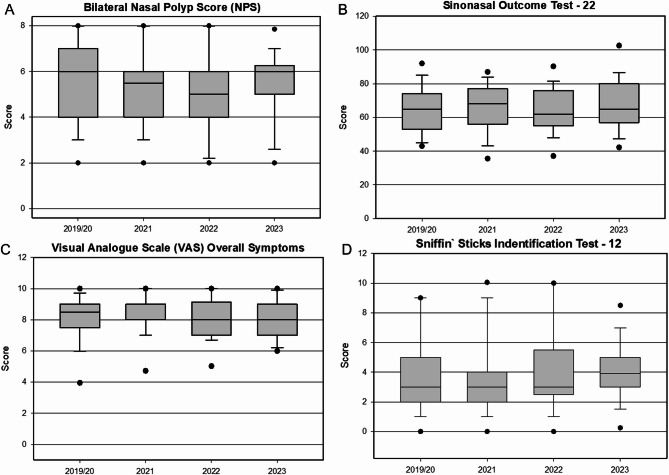
Boxplots comparing the values of (A) Bilateral Nasal Polyp Score, (B) Sinonasal Outcome Test − 22, (C) Visual Analogue Scale and (D) olfactory testing displayed on the scale of the Sniffin’ Sticks Identification Test − 12 between cohorts. Dots identifying 5–95% confidence interval.

Clinical parameters across the four cohorts showed some variability, but overall values were relatively similar, reflecting comparable baseline disease severity among cohorts as detailed in Table 2; Fig. 3.

**Table 2:** Clinical parameters measured before and during the treatment period within a real-world cohort of adult patients receiving dupilumab for chronic rhinosinusitis with nasal polyps (CRSwNP) separated into cohorts according to year of treatment initiation:

	2019/20		2021		2022		2023	
	0 months	6 months	0 months	6 months	0 months	6 months	0 months	6 months
NPS	5.7 (1.9)	1.2 (1.5)*	4.7 (1.7)	1.7 (2.1)*	5.2 (1.8)	1.1 (2.0)*	5.4 (1.5)	0.8 (2.1)*
SNOT-22	65.4 (14.5)	20.4 (14.0)*	67.7 (15.9)	34.7 (20.3)*	63.5 (14.1)	32.0 (19.6)*	68.5 (15.5)	26.7 (21.5)*
VAS	8.1 (1.6)	2.6 (1.8)*	8.3 (1.3)	3.3 (2.0)*	8.2 (1.3)	3.2 (1.7)*	8.1 (1.1)	2.2 (1.9)*
SSIT-12	3.8 (2.6)	7.9 (3.2)*	4.1 (1.8)	7.1 (2.9)*	4.1 (2.9)	6.0 (3.3)*	3.8 (2.1)	8.1 (2.2)*
**Category of olfactory function**								
Anosmia (%)	85.2	34.6	82.8	38.6	78.8	53.1	82.6	11.1
Hyposmia (%)	14.8	40.4	13.8	43.9	21.2	37.5	17.4	77.8
Normosmia (%)	0.0	25.0	3.5	17.6	0.0	9.4	0.0	11.1
Total IgE (U/l)	193.7 (221.0)	87.8 (167.5)	210.7 (246.8)*	46.0 (34.7)	144.4 (179.8)	64.7 (87.5)*	253.3 (355.0)	136.9 (179.1)*
Eosinophil count cells/µl	477.0 (410.1)	756.2 (372.7)*	332.7 (406.0)	612.1 (977.2)*	425.5 (347.8)	498.6	761.1 (1155.8)	873.1 (733.5)

Values are presented as the mean and standard deviation (in brackets), unless stated otherwise. Percentages provided are computed based on the proportion of patients with available data. *p < 0.05.

NPS, Nasal Polyp Score; SNOT-22, Sino-Nasal Outcome Test-22; VAS, Visual Analogue.

Scale for disease impairment; SSIT-12, Sniffin’ Sticks 12-item identification test; EUFOREA, European Forum for Research and Education in Allergy and Airway Diseases.

Baseline total NPS was slightly higher in the 2019/20 cohort at 5.7 ± 1.9, followed by 5.2 ± 1.8 in 2022, 4.7 ± 1.7 in 2021, and 5.4 ± 1.5 in 2023. While variation was minor, a modest dip in 2021 and 2022 suggests a possible trend toward earlier initiation of biologic therapy. SNOT-22 scores were comparable across cohorts, indicating similar symptom burden. Patients treated in 2019/20 had a baseline score of 65.4 ± 14.5, followed by 63.5 ± 14.1 in 2022, 67.7 ± 15.9 in 2021, and the highest score of 68.5 ± 15.5 in 2023. Although these values varied slightly, the differences were not statistically significant. VAS scores for overall disease burden remained consistently high across cohorts, with mean values of 8.1 ± 1.6 in 2019/20, 8.3 ± 1.3 in 2021, 8.2 ± 1.3 in 2022, and 8.1 ± 1.1 in 2023, suggesting uniform perception of disease severity regardless of treatment year. Olfactory function, assessed on a 0–12 scale, was comparably impaired across all years. The 2019/20 and 2023 cohorts both reported a mean baseline score of 3.8, with 4.1 in both 2021 and 2022. Most patients were anosmic at baseline: 85.2% in 2019/20, 82.8% in 2021, 78.8% in 2022, and 82.6% in 2023. Normosmic individuals were nearly absent across all groups. Total IgE levels varied by cohort, with 193.7 U/l ± 221.0 in 2019/20, 210.7 U/l ± 246.8 in 2021, 144.4 U/l ± 179.8 in 2022, and the highest value of 253.3 U/l ± 355.0 in 2023. Eosinophil counts showed modest fluctuations: 0.5 G/l ± 0.4 in 2019/20, 0.3 G/l ± 0.4 in 2021, 0.4 G/l ± 0.3 in 2022, and a peak at 0.8 G/l ± 1.1 in 2023. These values reflect overall stable markers of type 2 inflammation.

### Response to treatment

At the 6-month follow-up, all four cohorts exhibited substantial and significant improvements across key clinical parameters, though the magnitude of response varied slightly between cohorts.

The NPS showed consistent and statistically significant reductions across all cohorts (*p* < 0.05): 4.5 ± 0.3 points (2019/20), 3.0 ± 0.4 (2021), 4.1 ± 0.5 (2022), and 4.6 ± 0.6 (2023). These reductions exceeded the MCID of ≥ 1.0 point, indicating clinically meaningful improvements in polyp burden^23^. SNOT‑22 scores, reflecting symptom burden, also improved significantly (*p* < 0.05) in all cohorts, with reductions of 45.0 ± 2.7, 32.9 ± 3.4, 31.5 ± 4.4, and 41.8 ± 5.8, respectively—all well above the MCID of ≥ 8.9 points^23^. VAS scores for overall disease impairment decreased significantly (*p* < 0.05) by 5.5 ± 0.4, 5.1 ± 0.3, 5.0 ± 0.4, and 6.0 ± 0.5, respectively, exceeding the established MCID threshold of ≥ 1.0 point^23^. Olfactory function, assessed via SSIT‑12, showed significant improvement in all groups (*p* < 0.05), with mean increases of 4.1 ± 0.6, 3.0 ± 0.5, 1.9 ± 0.8, and 4.2 ± 0.7 points, respectively. These changes met or exceeded the MCID range for olfactory testing (2–4 points)^24^, indicating perceptible gains in smell function. Importantly, while all cohorts experienced meaningful clinical improvement, no cohort demonstrated a statistically superior response compared to the others.

## Discussion

While the efficacy and safety profile of dupilumab as a treatment for CRSwNP have been extensively documented in existing literature^13,15,25–27^, including recent work highlighting predictors of response and evolving patient profiles in clinical practice^28,29^ there remains limited evidence on how these factors have shifted over time. By analyzing consecutive yearly initiation cohorts, we assessed demographic trends, baseline disease severity, and treatment response, thereby providing insight into evolving realworld prescribing practices for CRSwNP.

While baseline demographics and overall disease severity were largely stable across cohorts, several notable trends emerged: a gradual decline in the proportion of patients with extensive surgical histories, consistent improvements in clinical outcomes across all years, and a marked reduction in new treatment initiations after 2021 despite stable eligibility profiles.

Demographically, mean age remained stable between 47.6 and 49.7 years, consistent with literature indicating peak CRSwNP prevalence between 40 and 60 years^6^. The 2023 cohort showed a reversal in gender predominance, with slightly more females than males; this shift was not statistically significant and may reflect subtle changes in referral patterns or recognition of CRSwNP in women. The prevalence of type 2 comorbidities remained high, with asthma and allergic rhinitis being the most frequent. While asthma prevalence dipped in 2021–2022 and rose again in 2023, allergic rhinitis showed a modest downward trend, underlining the persistent burden of type 2 inflammation in this population.

Baseline polyp size, measured by NPS, displayed minor, non‑significant variability. The slightly higher baseline NPS in 2019/20 could suggest that, early after dupilumab’s introduction, treatment was initiated in patients with more advanced disease. Subsequent years saw marginally lower scores, potentially reflecting earlier intervention as familiarity with the biologic increased. Olfactory dysfunction, a core symptom of CRSwNP, remained uniformly severe across all cohorts, without significant temporal trends.

The most striking clinical shift was the steady reduction in patients with extensive prior surgical histories. Those with > 3 prior ESS fell from 28.3% in 2019/20 to 13.0% in 2023, accompanied by decreases in the mean number of surgeries and extended intervals since the last ESS. These changes suggest a movement toward earlier biologic initiation, consistent with evolving treatment paradigms and the recommendations of EPOS 2020 and EUFOREA 2023^23^, which emphasize biologics for severe, surgeryrefractory CRSwNP.

Treatment outcomes were uniformly favorable. All cohorts experienced significant improvements in NPS, SNOT-22, VAS, and olfactory scores, with no statistically significant differences in the degree of improvement between years. This consistency reinforces dupilumab’s reproducible efficacy in realworld practice^15,25–27^. Variations in response magnitude are likely due to individual patient characteristics, such as immune profile and disease history, rather than intrinsic differences in drug effectiveness between cohorts.

While a detailed costeffectiveness analysis was outside the scope of this study, the high acquisition cost of dupilumab remains an important contextual factor in treatment selection. Prior analyses have demonstrated that, while biologics offer substantial clinical benefit, their costs far exceed those of surgical management in most health systems^30–32^. These economic realities likely contribute to the careful patient selection observed in our center, even as evidence for dupilumab’s efficacy grows.

However, this study has several limitations. It was conducted at a single tertiary referral center in Germany, which may limit generalizability to other regions or healthcare systems. The retrospective design also introduces the potential for selection bias. Additionally, external factors influencing prescribing trends—such as changes in outpatient referral patterns, insurance coverage policies, or evolving guideline interpretation—were not evaluated.

## Conclusion

In conclusion, our findings show that the demographic and clinical profiles of CRSwNP patients starting dupilumab have remained largely stable since 2019, with a trend toward initiating therapy in patients with fewer previous surgeries. Dupilumab produced significant and consistent improvements in both objective and patient‑reported outcomes across all yearly cohorts, with no significant differences in response. The decline in new initiations after 2021, despite similar eligibility profiles, indicates ongoing strict patient selection, likely reflecting adherence to EPOS 2020 recommendations and consideration of healthcare resource limitations in prescribing biologics for CRSwNP.

Author contributions:

PH designed the study, acquired, analyzed and interpreted all data and drafted the article. MG designed the study, was substantially involved in analysis and interpretation of data and in critically revising the article. CS and HF designed the study, helped to draft the manuscript and revised it critically. DG participated in the study design, acquisition and interpretation of data and revised the article critically. All authors gave final approval of the version to be published.

## Data Availability

Source data is available upon reasonable request from Patrick Huber.
